# Heart Rate Is a Better Predictor of Cardiorespiratory Fitness Than Heart Rate Variability in Overweight/Obese Children: The ActiveBrains Project

**DOI:** 10.3389/fphys.2019.00510

**Published:** 2019-05-07

**Authors:** Abel Plaza-Florido, Jairo H. Migueles, Jose Mora-Gonzalez, Pablo Molina-Garcia, Maria Rodriguez-Ayllon, Cristina Cadenas-Sanchez, Irene Esteban-Cornejo, Patricio Solis-Urra, Carlos de Teresa, Ángel Gutiérrez, Nathalie Michels, Jerzy Sacha, Francisco B. Ortega

**Affiliations:** ^1^PROFITH “PROmoting FITness and Health Through Physical Activity” Research Group, Department of Physical and Sports Education, Faculty of Sport Sciences, University of Granada, Granada, Spain; ^2^Department of Rehabilitation Sciences, KU Leuven – University of Leuven, Leuven, Belgium; ^3^Center for Cognitive and Brain Health, Department of Psychology, Northeastern University, Boston, MA, United States; ^4^IRyS Group, School of Physical Education, Pontificia Universidad Católica de Valparaíso, Valparaíso, Chile; ^5^Andalusian Centre of Sport Medicine (CAMD), Junta de Andalucía, Granada, Spain; ^6^Department of Medical Physiology, School of Medicine, University of Granada, Granada, Spain; ^7^Department of Public Health, Faculty of Medicine and Health Sciences, Ghent University, Ghent, Belgium; ^8^Faculty of Physical Education and Physiotherapy, Opole University of Technology, Opole, Poland; ^9^Department of Cardiology, University Hospital in Opole, University of Opole, Opole, Poland; ^10^Department of Biosciences and Nutrition, Karolinska Institutet, Huddinge, Sweden

**Keywords:** parasympathetic, sympathetic, heart rate variability, treadmill, adiposity, youth

## Abstract

Cardiac autonomic function can be quantified through mean heart rate (HR) or heart rate variability (HRV). Numerous studies have supported the utility of different HRV parameters as indicators of cardiorespiratory fitness (CRF). However, HR has recently shown to be a stronger predictor of CRF than HRV in healthy young adults, yet these findings need to be replicated, in other age groups such as children. Therefore, this study aimed: (1) to study the associations between indicators of cardiac autonomic function (HR, standard and corrected HRV parameters) and CRF in overweight/obese children; and (2) to test which of the two indicators (i.e., HR or HRV) is a stronger predictor of CRF. This study used cross-sectional baseline data of 107 overweight/obese children (10.03 ± 1.13 years, 58% boys) from the ActiveBrains project. Cardiac autonomic indicators were measured with Polar RS800CX^®^. CRF was assessed using a gas analyzer while performing a maximal incremental treadmill test. Correlations and stepwise linear regressions were performed. Mean HR and standard HRV parameters (i.e., pNN50, RMSSD, and SDNN) were associated with CRF (r coefficients ranging from -0.333 to 0.268; all *p* ≤ 0.05). The association of HR with CRF persisted after adjusting for sex, peak height velocity (PHV), adiposity moderate-to-vigorous physical activity, energy intake and circadian-related variable intradaily variability of activity patterns whilst for HRV parameters (i.e., pNN50, RMSSD, and SDNN) disappeared. Stepwise linear regression models entering HR and all HRV parameters showed that mean HR was the strongest predictor of CRF (β = -0.333, *R*^2^ = 0.111, *p* < 0.001). Standard and corrected HRV parameters did not provide additional value to the coefficient of determination (all *p* > 0.05). Our findings suggest that HR is the strongest indicator of CRF. It seems that quantification of HRV parameters in time and frequency domain do not add relevant clinical information about the cardiovascular health status (as measured by CRF) in overweight/obese children beyond the information already provided by the simple measure of HR.

## Introduction

A number of comorbidities linked to childhood obesity could be explained by their altered cardiac autonomic functions (Thayer and Lane, 2007; Zhou et al., 2012). Particularly, overweight/obese children and adults usually present a decreased parasympathetic activity (Gutin et al., 2000; Nagai et al., 2003; Rodríguez-Colón et al., 2011; Triggiani et al., 2017) which, in turn, is related to a higher risk of cardiovascular disease (CVD) (Lahiri et al., 2008). Therefore, the quantification of cardiac autonomic function is a matter of interest in overweight/obese populations.

Heart rate (HR) and heart rate variability (HRV) have been proposed as indicators of the cardiac autonomic nervous system functioning (Koizumi et al., 1985; Task Force, 1996; Lahiri et al., 2008). Specifically, HR reflects the frequency of heart beats per minute. HRV refers to the variability in time intervals between consecutive heart beats, namely between the R peaks registered in the electrocardiogram (ECG) (Task Force, 1996). Both HR and HRV have been used as predictors of CVD, showing that higher HR and lower HRV at rest are related to a higher CVD risk (Kannel et al., 1987; Thayer and Lane, 2007; Trimmel et al., 2015; Dilaveris and Tousoulis, 2018).

A good indicator of the CVD risk in adults is cardiorespiratory fitness (CRF) (Ortega et al., 2016; Ross et al., 2016; Tikkanen et al., 2017), which is also known as a powerful marker of health in children and adolescents (Ortega et al., 2008). In this context, HR is inversely associated with CRF (Kang et al., 2017), but based on a recent systematic review, associations between HRV and CRF are inconsistent in children and adolescents (Oliveira et al., 2017). For example, some studies have found positive associations between HRV and CRF in obese youth (Gutin et al., 2005; Michels et al., 2013; Da Silva et al., 2014), whereas others did not find differences in HRV parameters between 3 groups of healthy adolescents with different levels of CRF (Brunetto et al., 2005). In fact, Oliveira et al. concluded that several methodological differences between studies could contribute to these differences and that relevant studies in pediatric population are currently lacking.

Importantly, HRV reveals a non-linear inverse association with average HR (or direct association with average R-R interval), and consequently some clinical value of HRV must originate from average HR, – to elucidate this issue one should liberate HRV from the influence of HR (Sacha, 2013, 2014a; Sacha et al., 2013a; Sacha and Pluta, 2008). To overcome this HRV dependence on HR, a correction procedure has been proposed which is based on calculating the ratios between HRV parameters and different powers of their corresponding mean R-R interval (Sacha et al., 2013b). Of note, by employing this procedure, the HRV prediction capacity after myocardial infarction has been improved (Sacha, 2014b) and it was also possible to show that HR is a better predictor of CRF than HRV in young adults (Grant et al., 2013), although little is known on how it works in overweight/obese children.

Thus, the aims of this study were: (1) to explore the associations between indicators of cardiac autonomic function (HR, standard and corrected HRV parameters) and CRF; and (2) to test which of the two indicators (i.e., HR or HRV) is a stronger predictor of CRF in overweight/obese children.

## Materials and Methods

### Participants and Study Design

This cross-sectional study used baseline data from the ActiveBrains project^1^ (Clinical Trial: NCT02295072). A total of 107 overweight/obese children (10.03 ± 1.13 years, 58% boys) were included in the present study. Information about the methodology and protocol can be found elsewhere (Cadenas-Sánchez et al., 2016). This study was conducted according to the Declaration of Helsinki. The protocol was approved by the Committee for Research Involving Human Subjects at the University of Granada (Reference: 848, February 2014). All parents had received information about the study and gave written informed consent in accordance with the Declaration of Helsinki.

### Anthropometry and Weight Status

Body weight and height were measured to the nearest 0.1 kg and cm with an electronic scale and a stadiometer, respectively (SECA instruments, Germany, Ltd). Body mass index (BMI) was calculated as kg/m^2^ and the participants were classified as overweight, mild obesity or severe/morbid obesity according to the sex-and-age specific international BMI standards (World Obesity Federation, formerly named International Obesity Task Force) (Cole and Lobstein, 2012; Bervoets and Massa, 2014). Body fat percentage was assessed with dual-energy X-ray Absorptiometry (DXA, discovery densitometer from Hologic). All DXA scans and analyses were performed using the GE encore software (version 4.0.2) and were completed following the same protocol by the same evaluator. The positioning of the participants and the analyses of the results were undertaken following recommendations from the International Society of Clinical Densitometry (Crabtree et al., 2014).

### Physical Activity

Physical activity (PA) levels were estimated from the triaxial accelerometers ActiGraph GT3X+ (ActiGraph, Pensacola, FL, United States). Accelerometers were initialized to capture and store accelerations at 100 Hz and placed on non-dominant wrist for seven consecutive days. We processed the raw accelerations in the GGIR R package (Van Hees et al., 2013). We considered every participant wearing the accelerometer a minimum of 16 h per day during at least 3 weekdays and 1 weekend day as recommended elsewhere (Migueles et al., 2017, 2019). The daily time spent in moderate-to-vigorous PA intensity (i.e., >3 metabolic equivalents) was estimated using previously proposed cut-points for children (Hildebrand et al., 2014).

### Energy Intake

Energy intake was obtained by means of two non-consecutive 24-h recalls during a time span of a week by trained dietitians-nutritionists. The presence of parents or legal guardians was obligatory for the collection of dietary data due to the difficulty for children to remember recipes or amounts of foods. A book with pictures of different food servings and sizes was used to help the participants to estimate the amount of food consumed. The Diet software (Xyris Software, Brisbane, Australia), supported by the Spanish Association of Dietetics and Nutritionists, was used to calculated dietary data.

### Circadian-Related Variable Based on Fragmentation of Daytime Activity Patterns: Intradaily Variability

We calculated non-parametric indexes described by Van Someren et al. (1999) to characterize activity patterns. The circadian-related variable selected was fragmentation of daytime activity, measured by intradaily variability (IV), due to has been related to cardiorespiratory fitness and adiposity in adolescents (Garaulet et al., 2016). This circadian-related variable indicates the frequency of changes between high and low activity. Its values oscillated between 0, when the wave was perfectly sinusoidal, and 2, when the wave was as Gaussian noise.

### Heart Rate and Heart Rate Variability

Participants were placed supine for 10 min in a quiet and comfortable room between 9 a.m. and 12 p.m. Supine position has shown a higher reliability for the HRV measurement in children than sitting or standing (Silva et al., 2016). The POLAR RS800CX (Polar Electro Oy Inc., Kempele, Finland) recorded HRV during 10 min at a sampling frequency of 1000 Hz. This HR monitor is valid (Gamelin et al., 2008) and reliable when compared with electrocardiography (Vasconcellos et al., 2015) for the HRV assessment in children and adolescents. Participants were encouraged to keep relaxed, breathe normally and do not speak or move during the evaluation.

The indicators of cardiac autonomic function were HR and HRV parameters. For HRV analyses, we derived time and frequency-domain HRV parameters from the normal R-R intervals after excluding the extreme values using an automatized “low filter” in the Kubios software (HRV analysis, University of Eastern Finland) (Niskanen et al., 2004; Tarvainen et al., 2014). The R-R intervals series were detrended using the smoothness prior method with alpha set at 500 and a cubic interpolation at the default rate of 4 Hz. Out of the 10 min recorded, the middle 5 min (i.e., from 3 to 8 min) were visually checked for quality (i.e., normal distribution of the R-R intervals, no large R-R interval outliers and R-R intervals equidistance and minimal variation), and a different period of 5-min was selected when necessary. Time- and frequency-domain HRV parameters were selected based on the Guidelines of Task Force of The European Society of Cardiology and The North American Society of Pacing and Electrophysiology (Task Force, 1996). In the time-domain, we computed the squared root of the mean of the sum of the squares of successive normal R-R interval differences (RMSSD) and the percentage number of pairs of adjacent normal R-R intervals differing by more than 50 ms in the entire recording (pNN50), both as indexes of parasympathetic activity. We also computed the standard deviation of all normal R-R intervals (SDNN). In the frequency-domain, we performed spectral analyses using the non-parametric fast fourier transformation algorithm (FFT), with Welch’s periodogram method (i.e., 50% overlap Hanning window as pre-processing technique and calculating area under the curve with an integration). We derived total power of HRV spectrum (TP), the power in the high (HF) and the low frequency (LF) (TP: 0–0.4 Hz; HF: 0.15–0.4 Hz; LF: 0.04–0.15 Hz) in absolute units (ms^2^). HF and the LF/HF ratio were used in the analyses as indicators of parasympathetic activity and sympatho-vagal balance, respectively (Task Force, 1996), although physiological significance of LF and LF/HF ratio is not clear (Goldstein et al., 2011; Billman, 2013).

To remove the HRV dependence on HR, we calculated the corrected HRV parameters proposed by Sacha et al. (2013b) based in two assumptions: (i) If HRV parameters were negatively correlated with HR, correction procedure consisted in calculating ratios between HRV parameters and different powers of their corresponding mean normal R-R interval; (ii) if HRV parameters were positively correlated with HR, the correction procedure was performed by multiplying HRV parameters by the adequate powers of mean normal R-R interval as follows: RMMSD_c_ = RMSSD/meanRR^3.7^, pNN50_c_ = pNN50/meanRR^5.3^, SDNN_c_ = SDNN/meanRR^2.8^, FFT TP_c_ = TP/meanRR^6.3^, FFT HF_c_ = HF/meanRR^6.3^, FFT LF_c_ = LF/meanRR^5.0^, FFT LF/HF_c_ = LF/HF × meanRR^1.8^.

### Cardiorespiratory Fitness

Cardiorespiratory fitness was assessed using a gas analyzer (General Electric Corporation) while performing a maximal incremental treadmill test (HP-Cosmos ergometer) adapted for low-fit children (Davis et al., 2012). The incremental test consisted of walking on a treadmill at a constant speed (4.8 Km/h) starting at a 6% slope with grade increments of 1% every minute until volitional exhaustion. Children were highly encouraged to walk as long as possible. Maximal peak oxygen consumption (VO_2_peak, ml/kg/min), HR (beats/min) and respiratory exchange ratio (RER) were continuously recorded each 10 s, whilst the rating of perceived exertion (RPE) scale was reported at the end of each 1 min stage using children’s OMNI scale ranging from 0 to 10 (Suminski et al., 2008). VO2max was confirmed when meeting 3 out of 4 following criteria: achieving >85% of aged-predicted HRmax, a RER of ≥1.10, volitional fatigue (i.e., >8 points in the OMNI scale) and a plateau in the oxygen consumption during the last two exercise work rates (<2.0 ml/kg/min). We performed analysis with the complete sample that performed the treadmill test (VO_2_peak; *n* = 107) and with the sub-sample of children that met at least 3 out of 4 criteria described above (VO_2_max; *n* = 75). As conclusions were the same in both occasions, we presented here the results analyzing the complete sample with VO_2_peak (*n* = 107).

### Demographic Variables

Peak height velocity was assessed as an accurate and discriminant measure of maturational status (Malina et al., 2015). PHV was obtained from anthropometric variables (weight, height, and seated height) using Moore equations (Moore et al., 2015). Years from PHV offset were calculated by subtracting the age of PHV from the chronological age.

Socioeconomic status was assessed by the parental education level. Both father and mother self-reported their maximum educational level (i.e., elementary school, middle school, high school and university degree completed). Parents responses were then combined as follows: none of the parents had a university degree, one of the parents had a university degree or both parents had a university degree.

### Statistical Analyses

Descriptive data are presented as frequency and percentage for categorical variables. Kolmogorov-Smirnov test and a visual inspection of histograms were performed to assess the normality of data distribution. Variables that exhibited normal distribution were presented as mean and standard deviation (mean ± SD). Standard HRV parameters did not exhibit normal distribution and were presented as median and interquartile range. For analytical purpose, normal scores of standard and corrected HRV parameters were calculated using Blom formula (Blom, 1958).

In order to test the dependence between HRV and HR parameters, we performed Spearman correlations between standard and corrected HRV parameters and HR. First, the associations of HR, standard and corrected HRV parameters with CRF were studied using Bivariate Pearson correlations. Second, to determine the strongest predictors of CRF, we performed stepwise linear regressions of HR, standard and corrected HRV parameters with CRF. Predictors were included in two different stepwise models. The first model included HR and standard HRV parameters. The second model included HR and corrected HRV parameters. Lastly, we conducted exploratory analyses to test whether the observed associations were independent of potential confounders, such as sex, PHV, adiposity (body fat percentage), moderate-to-vigorous PA, energy intake and circadian-related variable. Analyses were performed using SPSS version 21.0 (IBM Corp., NY, United States). The significance level was set at 0.05.

## Results

**FIGURE 1:**
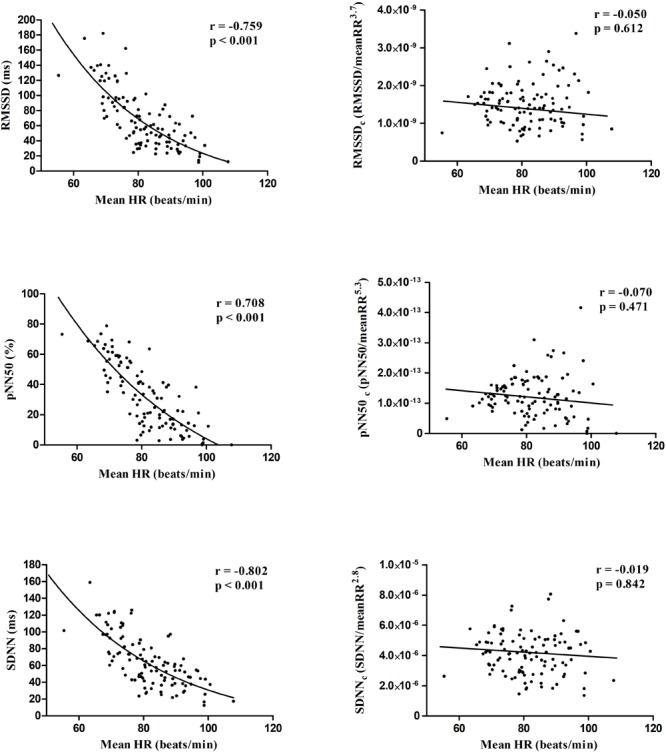
Scatter plots showing the Spearman correlations of time domain standard (left panels) and corrected (right panels) HRV parameters with mean heart rate.

Table 1 shows the descriptive characteristics of the participants. Figure 1, 2 present the Spearman correlations between HRV parameters and HR. Significant correlations found between the standard HRV parameters and HR (r ranging from -0.802 to 0.250; all *p* ≤ 0.009) disappeared after the correction procedure (r ranging from -0.120 to 0.033; *p* ≥ 0.220).

**Table 1:** Descriptive characteristics of the study sample (*n* = 107).

Variables	Total sample (*n* = 107)	Boys (*n* = 62, 58%)	Girls (*n* = 45, 42%)
**Parents with university degree, n (%)**					
None of them	71 (66.4)	45 (72.6)	26 (57.8)
One of them	19 (17.8)	9 (14.5)	10 (22.2)
Both of them	17 (15.9)	8 (12.9)	9 (20.0)
**Age and maturational status**
Age (years)	10.03 ± 1.13	10.15 ± 1.16	9.88 ± 1.08
PHV offset (years)	–2.26 ± 0.99	–2.65 ± 0.81	–1.71 ± 0.97
**Weight status**			
Weight (kg)	56.05 ± 11.06	56.86 ± 10.92	54.94 ± 11.28
Height (cm)	144.13 ± 8.36	144.85 ± 7.86	143.13 ± 8.99
BMI (kg/m^2^)	26.77 ± 3.62	26.92 ± 3.74	26.58 ± 3.47
BF (%)	44.50 ± 5.31	42.870 ± 5.04	45.65 ± 5.87
**Physical activity, energy intake, and circadian variable**			
Moderate-to-vigorous physical activity (min/day)	51.07 ± 19.85	58.66 ± 20.89	40.21 ± 11.72
Energy intake (Kcal/day)	1624.3 ± 430.77	1639.7 ± 433.17	1601.8 ± 431.39
Fragmentation (IV)	0.42 ± 0.05	0.41 ± 0.05	0.44 ± 0.05
**Cardiorespiratory fitness**			
VO2peak (ml/kg/min)	37.31 ± 4.73	37.71 ± 4.94	36.77 ± 4.14
**HR and HRV**			
Mean HR (bpm)	81.49 ± 9.71	80.4 ± 9.33	82.93 ± 10.12
Mean RR (ms)	746.90 ± 90.74	755.83 ± 87.3	734.60 ± 94.86
RMSSD (ms)	60.07 [56.51]	61.50 [51.06]	58.77 [49.22]
pNN50 (%)	30.94 [38.49]	33.59 [39.36]	26.87 [35.45]
SDNN (ms)	60.10 [42.44]	62.61 [47.82]	55.78 [37.09]
FFT TP (ms^2^)	3068.13 [5404.60]	3678.96 [5584.59]	2611.82 [3771.87]
FFT HF (ms^2^)	1222.92 [2641.93]	1410.64 [2945.46]	1142.54 [2316.98]
FFT LF (ms^2^)	1287.67 [1723.94]	1331.40 [1687.08]	1260.57 [1385.01]
FFT LF/HF (ms^2^)	0.92 [1.17]	0.92 [1.20]	0.91 [1.13]

*Data presented as mean ± SD, and as number and frequency. Median (IQR: interquartil range) are presented for standard HRV parameters because these variables presented skewed distributions. PHV, peak height velocity; BMI, body mass index; BF%, body fat; MVPA, moderate-to-vigorous physical activity; IV, intradaily variability; HR, heart rate; RR, R-R intervals; RMSSD, the square root of the mean of the sum of the squares of differences between adjacent NN intervals; pNN50, percentage (%) of the total pairs of RR intervals that differ by more than 50 ms; SDNN, standard deviation of all normal R-R intervals; TP, total power; FFT, fast fourier transformation; HF, high frequency band index of parasympathetic activity; LF, low frequency band mix index of sympathetic and parasympathetic activity.*

**FIGURE 2:**
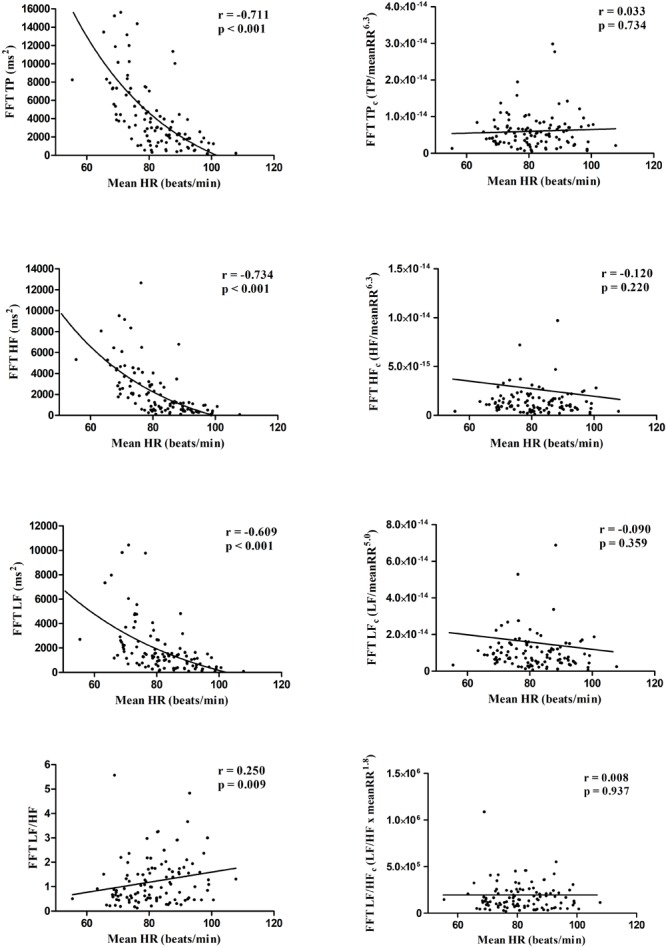
Scatter plots showing the Spearman correlations of frequency domain standard (left panels) and corrected (right panels) HRV parameters with mean heart rate.

Table 2 presents Pearson correlations of HR, and time and frequency normal scores (according to Blom, 1958) of standard and corrected HRV parameters with CRF. HR was inversely associated with CRF (*r* = -0.333, *p* < 0.001). The standard HRV parameters in time-domain (RMSSD, pNN50, and SDNN) were positively associated with CRF (r ranging from 0.209 to 0.268, all *p* < 0.050), whereas frequency-domain parameters were not significantly associated with CRF (*p* < 0.05). After correction for the prevailing HR, the RMSSD, pNN50 and SDNN completely lost their association with CRF, i.e., none of the corrected HRV parameter correlated with CRF (all *p* > 0.05).

**Table 2:** Pearson bivariate correlations of HR, standard and corrected HRV parameters with cardiorespiratory fitness (CRF) in overweight/obese children.

	Cardiorespiratory fitness, VO_2_peak (mL/kg/min)			
	HR and standard HRV parameters		Corrected HRV parameters	
	r	p	r	p
Mean HR (beats/minute)	**–0.333^∗^**	**<0.001**	Not applicable	Not applicable
				
RMSSD (ms)	**0.225**	**0.020**	**–**0.068	0.489
pNN50 (%)	**0.268**	**0.005**	**–**0.070	0.475
SDNN (ms)	**0.209**	**0.030**	**–**0.067	0.492
FFT TP (ms^2^)	0.157	0.107	–0.146	0.134
FFT HF (ms^2^)	0.174	0.073	–0.043	0.658
FFT LF (ms^2^)	0.080	0.415	–0.026	0.794
FFT LF/HF (ms^2^)	–0.164	0.092	–0.120	0.219

*^∗^*p* < 0.001. Bold numbers indicate *p* < 0.05.*

Table 3 shows stepwise linear regressions of HR and normal scores of standard and corrected HRV parameters with CRF. Out of all variables entered in the model, only HR was selected in the stepwise model as a significant predictor of CRF (*R*^2^ = 0.111, *p* < 0.001). The associations of HR with CRF remained significant (β = -0.169, *p* = 0.030), after adjustment for potential confounders (i.e., sex, PHV, adiposity [body fat percentage], moderate-to-vigorous PA, energy intake and circadian-related variable); whereas the associations of pNN50 and the rest of the HRV parameters with CRF became/remained non-significant (*p* > 0.05) (Supplementary Table S1).

**Table 3:** Stepwise linear regressions of HR, standard and corrected HRV parameters with cardiorespiratory fitness (CRF) in overweight/obese children.

Variables initially entered in the model	Significant predictors (*p* ≤ 0.05)	*p*-value	Standardized beta coefficient	Coefficient of determination R^2^
HR and standard HRV parameters: RMSSD, pNN50, SDNN, FFT TP, FFT HF, FFT LF, FFT LF/HF	HR	<0.001	–0.333	0.111
HR and corrected HRV parameters: RMSSD_c_, pNN50_c_, SDNN_c_, FFT TP_c_, FFT HF_c_, FFT LF_c_, FFT LF/HF_c_	HR	<0.001	–0.333	0.111

## Discussion

The main findings of this study were that: (1) HR showed stronger associations with CRF compared with standard and corrected HRV parameters in either time or frequency domains, regardless of sex, maturation, adiposity, moderate-to-vigorous PA, energy intake and circadian-related variable; (2) HR, was the only independent parameter associated with CRF when all the HRV parameters were included in the model. These results suggest that the complex concept of HRV does not seem to add clinically relevant information about the cardiovascular health status, as measured by CRF, in overweight/obese children beyond the information already provided by the “simple” measure of HR.

We found positive associations between standard HRV parameters in time domain (i.e., RMSSD, pNN50, and SDNN) and CRF, with pNN50 being the HRV parameter more strongly associated. In several studies, parasympathetic activity quantified through standard HRV parameters in time and frequency domains (i.e., RMSSD, pNN50, and HF) has been shown to be positively associated with CRF in children and obese adolescents (Gutin et al., 2005; Michels et al., 2013; Da Silva et al., 2014; Redón et al., 2016). However, a recent systematic review showed controversial associations between standard HRV parameters, used as indicators of parasympathetic activity, and CRF in children and adolescents (Oliveira et al., 2017). The authors concluded that the main limitations of most of the studies were the lack of a detailed explanation of HRV protocol, the use of different protocols to measure CRF and the lack of adjustment for relevant confounders (Oliveira et al., 2017).

In this context, it has been reported that HRV was significantly associated with CRF independently of age, race, sex and adiposity in adolescents (Gutin et al., 2005). However, in our study we show that the associations between HRV and CRF was not significant after controlling for potential confounders (sex, maturation, adiposity, moderate-to-vigorous physical activity, energy intake, circadian-related variable and HR). These contradictory findings can be explained by different factors: participants of different ranges of age (adolescents 14–18 years vs. overweight/obese children 8–11 years); use of different HRV protocol and instrument (ECG vs. HR monitor) and different methodology of CRF assessment (VO_2_ at HR of 170 bpm vs. maximal incremental treadmill test using a gas analyzer). Also, lack of studies in pediatric population studying associations between HRV and CRF should be considered (Oliveira et al., 2017), specifically in overweight/obese children.

To date, the majority of studies have often omitted the dependence of HRV on HR in overweight/obese children and adolescents (Rodríguez-Colón et al., 2011; Baum et al., 2013; Da Silva et al., 2014; Redón et al., 2016). This dependency might have significant influence on HRV since resting HR is different per each individual. In this sense, a correction procedure has been proposed which is based on ratios between the HRV parameters and different powers of their corresponding mean R-R intervals (Sacha et al., 2013b). In fact, the correction of HRV for prevailing HR is a kind of adjustment for HR at the individual level and it provides HRV parameters which are independent on HR. This method allows us to investigate the HR impact on the prognostic power of HRV in various clinical circumstances, e.g., this impact turned out to be positive for the prediction of cardiac death but negative for the prediction of non-cardiac death among patients after myocardial infarction (Sacha et al., 2013a). Moreover, in women, who usually present high HR, the exclusion of the HR impact on HRV improved the HRV prediction capability for all modes of death (Sacha et al., 2014). However, only one study has used the corrected parameters in relation to CRF in young adults and found that HR was a better predictor of CRF than the standard and corrected HRV parameters (Grant et al., 2013). In addition, they found that from all HRV indices, pNN50 was the most strongly associated with CRF, but HR was a better indicator of CRF than all the standard and corrected HRV parameters in young adults (Grant et al., 2013).

Our results in overweight/obese children are in line with the previous study in young adults (Grant et al., 2013). We found that some standard HRV parameters were associated with CRF, yet they lost this association after correction for HR, i.e., none of the corresponding corrected HRV parameter correlated with CRF (Table 2) – thus, HR seems to be a driving force in the association between standard HRV and CRF. The multiple regression analysis also confirmed that HR, but not HRV, is the only independent indicator of CRF. However, comparing with the study by Grant et al., our multiple stepwise regression revealed that only HR was a significant determinant (explained 11.1% of changes on CRF), but Grant et al. showed that both HR and HF were relevant (explained 17 and 3.1% respectively). Beyond the age range and weight status, the only methodological remarkable difference between our study and the previous one was that we measured CRF directly from gas analyzer (i.e., lab test) and Grant et al. (Grant et al., 2013) estimated CRF from the Cooper 12 min test (i.e., field test). Collectively, previous findings in young adults and present findings in overweight/obese children suggest that the associations between HRV and CRF exit mainly due to relationship between HR and CRF.

To our knowledge this is the first study showing that HR, as indicator of cardiac autonomic function, is a stronger predictor of CRF than HRV parameters in time and frequency domain in overweight/obese children. Therefore, it seems that the more complex concept of HRV do not provide additional information compared with the HR measurement in children with weight disturbances. This may have an important practical consequence, i.e., therapeutic interventions in such children should be directed at the reduction of HR which is an easy parameter and provides valuable information on CRF. However, future studies should perform these analyses in other populations of different age ranges (i.e., older adults) and health status (i.e., healthy children, obese adults, older adults with cardiovascular diseases, etc.).

### Strengths and Limitations

Several limitations need to be acknowledged: (1) since our study design is cross-sectional, we cannot assume a causal relationship; (2) we did not use a gold standard for the HRV measurement, however, the RS800CX has demonstrated to be valid (Gamelin et al., 2008) and reliable (Vasconcellos et al., 2015) for this measurement; (3) some studies have found HRV parameters to be affected by breathing (Wessel et al., 2009; Sidorenko et al., 2016), but this effect is not clear and some parameters can be less affected by changes in respiration (Hill and Siebenbrock, 2009), so we decided not to control the breathing to avoid disturbing the resting status of the participants; (4) HRV analyses were performed only in time and frequency domain, maybe other complex approaches to analyze HRV (i.e., approximate and sample entropies, detrended fluctuation analyses short term exponent, etc.,) (Sassi et al., 2015) could add additional information in relation to the prediction of CRF. The main strengths of our study were: (1) CRF was objectively measured with a gas analyzer (gold standard), using an adapted protocol recommended to low-fit children and (2) to the best of our knowledge this is the first study confirming that HR is a better predictor of CRF than complex HRV parameters in overweight/obese children.

## Conclusion

In conclusion, HR is a better predictor of CRF than HRV parameters, in either time or frequency domains, in overweight/obese children. Further, HRV parameters did not add any significant information to the models in our overweight/obese participants. Thus, the complex concept of HRV might not provide additional information to the prediction of CRF. Clinicians and sport scientists that use cardiac autonomic activity as indicator of cardiovascular health represented by CRF should take into consideration that the “simple” HR metric gives more information about CRF levels of overweight/obese children than any HRV parameter. Furthermore, the HR assessment and its interpretation are markedly easier than the ones of HRV parameters.

## Ethics Statement

This study was conducted according to the Committee for Research Involving Human Subjects at the University of Granada (Reference: 848, February 2014).

## Author Contributions

JM, J M-G, CC-S, and FO contributed to design the experiment and study design. AP-F, JM, JM-G, PM-G, MR-A, CC-S, IE-C, JS, and FO contributed to acquisition, analysis, or interpretation of data for the manuscript. All authors drafted or critically revised the manuscript for important intellectual content and approved the final version of the manuscript to be published.

## Conflict of Interest Statement

The authors declare that the research was conducted in the absence of any commercial or financial relationships that could be construed as a potential conflict of interest.

## Footnotes

1 http://profith.ugr.es/activebrains?lang=en
