# Supplementary material for: Heart Rate Is a Better Predictor of Cardiorespiratory Fitness Than Heart Rate Variability in Overweight/Obese Children: The ActiveBrains Project
Source: Front Physiol. 2019 May 7;10:510. doi: 10.3389/fphys.2019.00510 (PMC6514130; doi:10.3389/fphys.2019.00510)
Supplement: Supplementary file 1 [file Table_1.DOCX]

|  | Cardiorespiratory fitness, VO_2_peak (mL/kg/min) | |
| --- | --- | --- |
|  | Standardized beta coefficient | p value |
| Model 1 Adjusted by potential confouderns: sex, PHV, adiposity (BFP%). | | |
| Mean HR (beats/minute) | **-0.164** | **0.029** |
| RMSSD (ms) | 0.081 | 0.258 |
| pNN50 (%) | 0.098 | 0.178 |
| SDNN (ms) | 0.062 | 0.388 |
| Model 2 Adjusted by potential confouderns: sex, PHV, adiposity (BFP%) and MVPA. | | |
| Mean HR (beats/minute) | **-0.156** | **0.040** |
| RMSSD (ms) | 0.076 | 0.295 |
| pNN50 (%) | 0.094 | 0.201 |
| SDNN (ms) | 0.058 | 0.424 |
| Model 3 Adjusted by potential confouderns: sex, PHV, adiposity (BFP%), MVPA and, energy intake. | | |
| Mean HR (beats/minute) | **-0.173** | **0.029** |
| RMSSD (ms) | 0.068 | 0.372 |
| pNN50 (%) | 0.088 | 0.254 |
| SDNN (ms) | 0.056 | 0.470 |
| Model 4 Adjusted by potential confouderns: sex, PHV, adiposity (BFP%), MVPA, energy intake and circadian-related variable (IV) | | |
|  |  |  |
| Mean HR (beats/minute) | **-0.169** | **0.030** |
| RMSSD (ms) | 0.065 | 0.391 |
| pNN50 (%) | 0.079 | 0.298 |
| SDNN (ms) | 0.044 | 0.565 |

**Table S1.** Linear regression models on the associations of HR and normal scores of standard HRV parameters with cardiorespiratory fitness (CRF).
